# 2,2′-{1,1′-[Pentane-1,5-diyl­bis(oxy­nitrilo)]­diethyl­idyne}diphenol

**DOI:** 10.1107/S160053680803136X

**Published:** 2008-10-04

**Authors:** Wen-Kui Dong, Zhong-Wu Lv, Xue-Ni He, Yong-Hong Guan, Jun-Feng Tong

**Affiliations:** a School of Chemical and Biological Engineering, Lanzhou Jiaotong University, Lanzhou 730070, People’s Republic of China

## Abstract

In the title compound, C_21_H_26_N_2_O_4_, there is half a mol­ecule in the asymmetric unit with a crystallographic twofold rotation axis passing through the central C atom of the –CH=N—O—(CH_2_)_5_—O—N=CH– bridge. The dihedral angle formed by the two benzene rings is 80.85 (2)°. Strong intra­molecular O—H⋯N and C—H⋯O hydrogen bonds help to establish the molecular conformation. There are also weak inter­molecular π–π stacking inter­actions between neighbouring benzene rings [centroid–centroid separation = 3.502 (3) Å].

## Related literature

For general background, see: Bhadbhade & Srinivas (1993 ▶). For related structures, see: Dong *et al.* (2007 ▶, 2008 ▶); Wang *et al.* (2007 ▶); Xu *et al.* (2007 ▶).
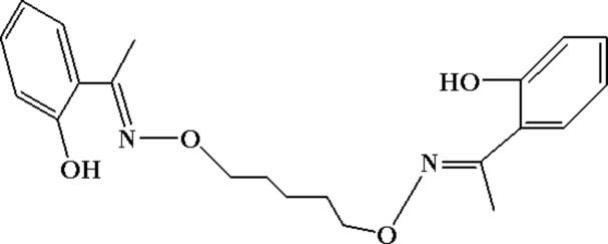


## Experimental

### 

#### Crystal data

C_21_H_26_N_2_O_4_
*M* *_r_* = 370.44
Monoclinic, 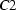

*a* = 12.9691 (13) Å
*b* = 4.601 (1) Å
*c* = 16.3639 (16) Å
β = 91.621 (1)°
*V* = 976.1 (3) Å^3^
*Z* = 2
Mo *K*α radiation
μ = 0.09 mm^−1^
*T* = 298 (2) K
0.48 × 0.40 × 0.32 mm

#### Data collection

Siemens SMART 1000 CCD area-detector diffractometer
Absorption correction: multi-scan (*SADABS*; Sheldrick, 1996 ▶) *T*_min_ = 0.959, *T*_max_ = 0.973
2421 measured reflections
972 independent reflections
646 reflections with *I* > 2σ(*I*)
*R*_int_ = 0.038

#### Refinement

*R*[*F* ^2^ > 2σ(*F* ^2^)] = 0.042
*wR*(*F* ^2^) = 0.123
*S* = 1.04
972 reflections
123 parameters
2 restraints
H-atom parameters constrained
Δρ_max_ = 0.15 e Å^−3^
Δρ_min_ = −0.12 e Å^−3^

### 

Data collection: *SMART* (Siemens, 1996 ▶); cell refinement: *SAINT* (Siemens, 1996 ▶); data reduction: *SAINT*; program(s) used to solve structure: *SHELXS97* (Sheldrick, 2008 ▶); program(s) used to refine structure: *SHELXL97* (Sheldrick, 2008 ▶); molecular graphics: *SHELXTL* (Sheldrick, 2008 ▶); software used to prepare material for publication: *SHELXTL*.

## Supplementary Material

Crystal structure: contains datablocks global, I. DOI: 10.1107/S160053680803136X/gw2048sup1.cif

Structure factors: contains datablocks I. DOI: 10.1107/S160053680803136X/gw2048Isup2.hkl

Additional supplementary materials:  crystallographic information; 3D view; checkCIF report

## Figures and Tables

**Table 1:** Hydrogen-bond geometry (Å, °)

*D*—H⋯*A*	*D*—H	H⋯*A*	*D*⋯*A*	*D*—H⋯*A*
O2—H2⋯N1	0.82	1.84	2.553 (4)	144
C4—H4*A*⋯O1	0.96	2.16	2.631 (5)	109

**Fig. 1.**
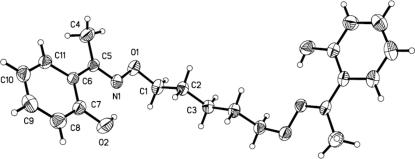
The molecular structure of the title compound with atom numbering scheme [Symmetry codes: A: -x, y, -z + 1]. Displacement ellipsoids for non-hydrogen atoms are drawn at the 30% probability level.

## supplementary crystallographic information

### Comment

salen-type compounds containing strong donor sites such as oxygen and imine nitrogen atoms and their metal complexes have been the subject of extensive investigation (Bhadbhade & Srinivas, 1993). Structures of salen-type compounds derived from *O*-alkyl oxime moiety (–CH═N—O-(CH_2_)_n_-O—N═CH–) instead of the imine moiety and closely related to the title compound (Wang *et al.*, 2007; Dong *et al.*, 2008; Dong *et al.*, 2007), are known. In this research, we report the synthesis and crystal structure of the title compound, and shown in Fig. 1.

The single-crystal structure of the title compound is built up by discrete C_21_H_26_N_2_O_4_ molecules, within all bond lengths are in normal ranges. There is 1/2 molecule per asymmetric unit with a crystallographic twofold rotation axis passing through the central carbon (symmetry code: -*x*, *y*, -*z*) of the five carbon atoms in the (—CH═N—O—(CH_2_)_5_—O—N═CH—) bridge. The dihedral angle formed by the two benzene rings in the molecule of the title compound is 80.85 (2)°. The strong intramolecular O2—H2···N1 and C4—H4A···O1 hydrogen bonds play an important role in the stability of the crystal structure in the title compound. The five carbon atoms in the C1—C2—C3—C2A—C1A bridge are almost in the same plane with slight deviation of 0.015 and 0.029 Å below for C1, C2A, 0.015 and 0.029 Å above the plane for C2 and C1A (symmetry code A: -*x*, *y*, -*z* + 1), respectively. In the crystal structure, there is weak intermolecular π–π stacking interaction between the neighbouring benzene rings, and the inter-molecular plane-to-plane distance is 3.502 (3) Å along *b* axis. This structure is not similar to what was observed in our previously reported series of salen-type compound containing two- (Wang *et al.*, 2007), three- (Dong *et al.*, 2008) and four-methene (Dong *et al.*, 2007) bridge.

### Experimental

2,2'-[1,1'-(Pentane-1,5-diyldioxydinitrilo)diethylidyne]diphenol was synthesized according to an analogous method reported earlier (Wang *et al.*, 2007; Xu *et al.*, 2007). To an ethanol solution (5 ml) of 2'-hydroxyacetophenone (516.2 mg, 4.00 mmol) was added an ethanol solution (3 ml) of 1,5-bis(aminooxy)pentane (268.4 mg, 2.00 mmol). The reaction mixture was stirred at 328 K for 4 h. The formed precipitate was separated by filtration, and washed successively with ethanol and ethanol–hexane (1:4), respectively. The product was dried under vacuum to yield 410.9 mg of the title compound. Yield, 55.5%. mp. 344–345 K. Anal. Calc. for C_17_H_16_Cl_2_N_2_O_2_: C, 68.09; H, 7.07; N, 7.56. Found: C, 68.19; H, 7.21; N, 7.42.

Colorless block-like single crystals suitable for X-ray diffraction studies were obtained after two weeks by slow evaporation from a diethyl-ether solution of the title compound.

### Refinement

Non-H atoms were refined anisotropically. H atoms were treated as riding atoms with distances C—H = 0.97 (CH_2_), 0.93 Å (CH), O—H = 0.82 Å and *U*_iso_(H) = 1.2 *U*_eq_(C) and 1.5 *U*_eq_(O).

### Crystal data

**Table:**

C_21_H_26_N_2_O_4_	*F*(000) = 396
*M**_r_* = 370.44	*D*_x_ = 1.260 Mg m^−^^3^
Monoclinic, *C*2	Mo *K*α radiation, λ = 0.71073 Å
Hall symbol: C 2y	Cell parameters from 813 reflections
*a* = 12.9691 (13) Å	θ = 2.5–23.2°
*b* = 4.601 (1) Å	µ = 0.09 mm^−^^1^
*c* = 16.3639 (16) Å	*T* = 298 K
β = 91.621 (1)°	Needle-like, colourless
*V* = 976.1 (3) Å^3^	0.48 × 0.40 × 0.32 mm
*Z* = 2	

### Data collection

**Table:**

Siemens SMART 1000 CCD area-detector diffractometer	972 independent reflections
Radiation source: fine-focus sealed tube	646 reflections with *I* > 2σ(*I*)
graphite	*R*_int_ = 0.038
φ and ω scans	θ_max_ = 25.0°, θ_min_ = 2.5°
Absorption correction: multi-scan (SADABS; Sheldrick, 1996)	*h* = −15→15
*T*_min_ = 0.959, *T*_max_ = 0.973	*k* = −5→5
2421 measured reflections	*l* = −19→10

### Refinement

**Table:**

Refinement on *F*^2^	Primary atom site location: structure-invariant direct methods
Least-squares matrix: full	Secondary atom site location: difference Fourier map
*R*[*F*^2^ > 2σ(*F*^2^)] = 0.042	Hydrogen site location: inferred from neighbouring sites
*wR*(*F*^2^) = 0.123	H-atom parameters constrained
*S* = 1.04	*w* = 1/[σ^2^(*F*_o_^2^) + (0.0568*P*)^2^ + 0.2844*P*] where *P* = (*F*_o_^2^ + 2*F*_c_^2^)/3
972 reflections	(Δ/σ)_max_ < 0.001
123 parameters	Δρ_max_ = 0.15 e Å^−^^3^
2 restraints	Δρ_min_ = −0.12 e Å^−^^3^

### Special details

**Table:**

Geometry. All e.s.d.'s (except the e.s.d. in the dihedral angle between two l.s. planes) are estimated using the full covariance matrix. The cell e.s.d.'s are taken into account individually in the estimation of e.s.d.'s in distances, angles and torsion angles; correlations between e.s.d.'s in cell parameters are only used when they are defined by crystal symmetry. An approximate (isotropic) treatment of cell e.s.d.'s is used for estimating e.s.d.'s involving l.s. planes.
Refinement. Refinement of *F*^2^ against ALL reflections. The weighted *R*-factor *wR* and goodness of fit *S* are based on *F*^2^, conventional *R*-factors *R* are based on *F*, with *F* set to zero for negative *F*^2^. The threshold expression of *F*^2^ > σ(*F*^2^) is used only for calculating *R*-factors(gt) *etc*. and is not relevant to the choice of reflections for refinement. *R*-factors based on *F*^2^ are statistically about twice as large as those based on *F*, and *R*- factors based on ALL data will be even larger.

### Fractional atomic coordinates and isotropic or equivalent isotropic displacement parameters (Å^2^)

**Table:**

	*x*	*y*	*z*	*U*_iso_*/*U*_eq_	Occ. (<1)
N1	0.1857 (2)	0.7552 (7)	0.30501 (17)	0.0533 (8)	
O1	0.23002 (19)	0.6005 (8)	0.37063 (14)	0.0656 (7)	
O2	0.0337 (2)	0.9262 (9)	0.21462 (17)	0.0796 (9)	
H2	0.0629	0.8223	0.2488	0.119*	
C1	0.1512 (3)	0.4342 (11)	0.4082 (2)	0.0607 (10)	
H1A	0.1834	0.2927	0.4447	0.073*	
H1B	0.1126	0.3286	0.3662	0.073*	
C2	0.0778 (3)	0.6170 (10)	0.4558 (2)	0.0551 (9)	
H2A	0.1169	0.7333	0.4952	0.066*	
H2B	0.0418	0.7489	0.4186	0.066*	
C3	0.0000	0.4391 (13)	0.5000	0.0526 (12)	
H3A	0.0361	0.3147	0.5392	0.063*	0.50
H3B	−0.0361	0.3147	0.4608	0.063*	0.50
C4	0.3610 (3)	0.9300 (15)	0.2926 (2)	0.0836 (14)	
H4A	0.3736	0.8041	0.3386	0.125*	
H4B	0.3787	1.1259	0.3074	0.125*	
H4C	0.4023	0.8688	0.2479	0.125*	
C5	0.2494 (3)	0.9162 (9)	0.2673 (2)	0.0494 (9)	
C6	0.2084 (3)	1.0860 (9)	0.1985 (2)	0.0485 (9)	
C7	0.1043 (3)	1.0870 (11)	0.1759 (2)	0.0573 (9)	
C8	0.0682 (3)	1.2544 (12)	0.1116 (2)	0.0745 (12)	
H8	−0.0017	1.2535	0.0971	0.089*	
C9	0.1348 (4)	1.4212 (13)	0.0693 (2)	0.0773 (13)	
H9	0.1099	1.5343	0.0260	0.093*	
C10	0.2363 (4)	1.4250 (12)	0.0892 (2)	0.0735 (12)	
H10	0.2812	1.5389	0.0595	0.088*	
C11	0.2732 (3)	1.2605 (10)	0.1533 (2)	0.0610 (11)	
H11	0.3433	1.2656	0.1670	0.073*	

### Atomic displacement parameters (Å^2^)

**Table:**

	*U*^11^	*U*^22^	*U*^33^	*U*^12^	*U*^13^	*U*^23^
N1	0.0547 (18)	0.0514 (18)	0.0545 (16)	0.0036 (18)	0.0125 (14)	−0.0018 (17)
O1	0.0574 (15)	0.0702 (16)	0.0701 (15)	0.0067 (17)	0.0167 (12)	0.0136 (17)
O2	0.0532 (16)	0.0868 (19)	0.099 (2)	−0.006 (2)	0.0049 (14)	0.018 (2)
C1	0.065 (2)	0.053 (2)	0.066 (2)	0.011 (3)	0.0173 (19)	0.009 (2)
C2	0.062 (2)	0.045 (2)	0.059 (2)	−0.001 (2)	0.0143 (17)	−0.002 (2)
C3	0.062 (3)	0.041 (3)	0.054 (3)	0.000	0.009 (2)	0.000
C4	0.055 (2)	0.107 (4)	0.089 (3)	−0.014 (3)	0.004 (2)	0.015 (4)
C5	0.0469 (19)	0.048 (2)	0.0541 (19)	−0.003 (2)	0.0155 (17)	−0.010 (2)
C6	0.053 (2)	0.0447 (19)	0.0478 (18)	−0.011 (2)	0.0105 (16)	−0.010 (2)
C7	0.056 (2)	0.055 (2)	0.062 (2)	−0.004 (3)	0.0132 (18)	−0.005 (3)
C8	0.071 (3)	0.078 (3)	0.074 (3)	−0.001 (3)	−0.004 (2)	0.005 (3)
C9	0.106 (4)	0.067 (3)	0.058 (2)	0.001 (3)	0.000 (2)	0.001 (3)
C10	0.098 (4)	0.066 (3)	0.058 (2)	−0.023 (3)	0.014 (2)	−0.004 (3)
C11	0.063 (2)	0.062 (3)	0.059 (2)	−0.014 (3)	0.013 (2)	−0.012 (3)

### Geometric parameters (Å, °)

**Table:**

N1—C5	1.282 (4)	C4—H4A	0.9600
N1—O1	1.398 (4)	C4—H4B	0.9600
O1—C1	1.430 (5)	C4—H4C	0.9600
O2—C7	1.349 (5)	C5—C6	1.459 (5)
O2—H2	0.8200	C6—C7	1.389 (5)
C1—C2	1.504 (5)	C6—C11	1.390 (5)
C1—H1A	0.9700	C7—C8	1.375 (5)
C1—H1B	0.9700	C8—C9	1.360 (6)
C2—C3	1.501 (5)	C8—H8	0.9300
C2—H2A	0.9700	C9—C10	1.348 (6)
C2—H2B	0.9700	C9—H9	0.9300
C3—C2^i^	1.501 (5)	C10—C11	1.370 (6)
C3—H3A	0.9700	C10—H10	0.9300
C3—H3B	0.9700	C11—H11	0.9300
C4—C5	1.495 (5)		
			
C5—N1—O1	114.0 (3)	C5—C4—H4C	109.5
N1—O1—C1	108.6 (3)	H4A—C4—H4C	109.5
C7—O2—H2	109.5	H4B—C4—H4C	109.5
O1—C1—C2	113.2 (4)	N1—C5—C6	117.0 (3)
O1—C1—H1A	108.9	N1—C5—C4	121.6 (4)
C2—C1—H1A	108.9	C6—C5—C4	121.3 (4)
O1—C1—H1B	108.9	C7—C6—C11	117.0 (4)
C2—C1—H1B	108.9	C7—C6—C5	122.5 (3)
H1A—C1—H1B	107.7	C11—C6—C5	120.4 (3)
C3—C2—C1	112.9 (3)	O2—C7—C8	116.4 (4)
C3—C2—H2A	109.0	O2—C7—C6	122.7 (4)
C1—C2—H2A	109.0	C8—C7—C6	120.8 (4)
C3—C2—H2B	109.0	C9—C8—C7	119.9 (4)
C1—C2—H2B	109.0	C9—C8—H8	120.1
H2A—C2—H2B	107.8	C7—C8—H8	120.1
C2—C3—C2^i^	113.9 (5)	C10—C9—C8	121.0 (5)
C2—C3—H3A	108.8	C10—C9—H9	119.5
C2^i^—C3—H3A	108.8	C8—C9—H9	119.5
C2—C3—H3B	108.8	C9—C10—C11	119.7 (4)
C2^i^—C3—H3B	108.8	C9—C10—H10	120.2
H3A—C3—H3B	107.7	C11—C10—H10	120.2
C5—C4—H4A	109.5	C10—C11—C6	121.6 (4)
C5—C4—H4B	109.5	C10—C11—H11	119.2
H4A—C4—H4B	109.5	C6—C11—H11	119.2
			
C5—N1—O1—C1	179.0 (3)	C5—C6—C7—O2	−1.6 (6)
N1—O1—C1—C2	−72.2 (4)	C11—C6—C7—C8	0.0 (6)
O1—C1—C2—C3	−175.9 (3)	C5—C6—C7—C8	178.7 (4)
C1—C2—C3—C2^i^	−176.9 (4)	O2—C7—C8—C9	−179.6 (4)
O1—N1—C5—C6	−179.5 (3)	C6—C7—C8—C9	0.1 (6)
O1—N1—C5—C4	0.5 (5)	C7—C8—C9—C10	0.2 (7)
N1—C5—C6—C7	2.9 (5)	C8—C9—C10—C11	−0.5 (8)
C4—C5—C6—C7	−177.1 (4)	C9—C10—C11—C6	0.6 (7)
N1—C5—C6—C11	−178.5 (3)	C7—C6—C11—C10	−0.3 (6)
C4—C5—C6—C11	1.5 (6)	C5—C6—C11—C10	−179.1 (4)
C11—C6—C7—O2	179.7 (4)		

Symmetry codes: (i) −*x*, *y*, −*z*+1.

### Hydrogen-bond geometry (Å, °)

**Table:**

*D*—H···*A*	*D*—H	H···*A*	*D*···*A*	*D*—H···*A*
O2—H2···N1	0.82	1.84	2.553 (4)	144
C4—H4A···O1	0.96	2.16	2.631 (5)	109
